# Impact of feeding a high fiber diet and roughage on stress and clinical welfare indicators in fast-growing broiler breeder pullets

**DOI:** 10.1016/j.psj.2025.105960

**Published:** 2025-10-08

**Authors:** Kaitlin E. Wurtz, Karen Thodberg, Anja B. Riber

**Affiliations:** Department of Animal and Veterinary Sciences, Aarhus University DK-8830 Tjele, Denmark

**Keywords:** Broiler breeder, Welfare, Qualitative feed restriction, Fault bar

## Abstract

Qualitative feed restriction has been proposed as a means to alleviate hunger, frustration, and stress associated with feed restriction in broiler breeder chickens. The aim of this study was to investigate the effect of a fiber-rich diet containing oat-hulls and a daily allocation of roughage (EXP) compared to a standard commercial diet (CON) on broiler breeder welfare. A total of 600 day-old female Ross 308 breeder chicks were allocated to 12 pens of 50 birds each, with each pen receiving one of the two dietary treatments. At the conclusion of the study, a welfare assessment was performed which included assessing plumage damage, wounds/scratches, footpad dermatitis, bumblefoot, hock burns, plumage cleanliness, and body weight. Wing, scapula, and tail feathers were collected and examined following euthanasia for presence and severity of fault bars. Litter quality was assessed visually every other week throughout the study period, and samples were collected at weeks 6, 12, and 18 to assess moisture content. Mortality was also documented throughout the study. Body weight uniformity, litter quality, and mortality did not differ between treatments. CON birds had poorer plumage quality (*P* < 0.0001), greater occurrence of hyperkeratosis (*P* < 0.0001), were more likely to have poorer hock burn scores (*P* = 0.0104) and were more likely to have dirtier plumage (*P* = 0.0075) than birds in the EXP treatment. CON birds had a greater number of fault bars (*P* < 0.0001) and greater occurrence of severe fault bars (*P=* 0.039) on their scapula feathers than EXP birds. Feather growth rate was also lower in the CON treatment for scapula feathers (*P* = 0.0305). In conclusion, results from our study show that feeding a diet high in insoluble fiber and supplemented with roughage appears to provide some improvements to broiler breeder welfare during rearing compared to feeding a standard commercial diet.

## Introduction

Broiler breeders (the parents of broiler chickens) are commonly feed restricted. While this practice helps promote health and reproductive performance, it comes with challenges including poor welfare arising from hunger, frustration, and stress (Decuypere et al., 2010). Restriction is often most severe during the rearing period, prior to reaching sexual maturity (De Jong and Guémené, 2011). Qualitative feed restriction, in the form of bulking up the feed with fiber, has been proposed as a means to increase the amount of feed that birds may consume to increase the sensation of satiation without substantially increasing energy intake.

Increases in injurious pecking may arise in response to increased hunger or decreased foraging opportunities, leading to plumage and skin damage (Girard, et al., 2017; Morrissey, et al., 2014). Plumage condition may also be negatively impacted by insufficient dietary protein (van Emous, et al., 2014), and nutritional deficiencies may trigger outbreaks of cannibalism (Rodenburg, et al., 2008), further contributing to tissue and plumage damage. Presence and severity of fault bars, bands of deficient keratin deposition across the feather, have been proposed as a measure of the response to acute stress in broiler breeders (Arrazola and Torrey, 2019). These weakened points in the feather are more prone to breakage (Jovani and Diaz‐Real, 2012) and can be another contributing factor to poor plumage condition.

Polydipsia (excessive thirst) may occur in conjunction with hunger, causing birds to consume greater amounts of water (Savory and Maros, 1993). Additionally, frustration may lead to increased displacement pecking towards water nipples, leading to increased spillage of water onto the litter (Duncan and Wood-Gush, 1972). Increases in water spillage, as well as a higher moisture content in the excreta, can contribute to poor litter condition. High litter moisture content increases the risk of contact dermatitis on footpads and hocks (Ekstrand, et al., 1997, 1998). Under feed restriction, birds must compete for access to feed, placing subordinate or weaker birds at risk of becoming underweight or victims of aggressive interactions from flockmates. Poor body weight uniformity has been shown to be associated with higher mortality rates and less efficient growth in broiler chickens (Vasdal, et al., 2019).

Previous work conducted by our group has shown that inclusion of maize silage (roughage) contributed to improved stress and clinical welfare indicators (Tahamtani, et al., 2020) and that feeding a diet diluted with oat hulls (insoluble fiber) lead to reduced frustration, reduced feeding motivation, and lower compensatory feed intakes, suggesting reduced hunger (Riber and Tahamtani, 2020). While qualitative feed restriction is regarded as a promising method of improving behavioral opportunities and welfare in broiler breeders, the optimal diet formulation remains to be determined (Taylor, et al., 2024a). The present study builds upon knowledge gained from the former studies as well as the latest findings in the literature. The aim of the present study was to investigate the effect of a fiber-rich diet containing oat-hulls and daily allocation of roughage on stress and clinical welfare indicators in broiler breeder pullets during the rearing period.

## Materials and methods

The experiment was carried out according to the guidelines of the Danish Animal Experiments Inspectorate with respect to animal experimentation and care of animals under study.

### Animals and housing

This study encompasses a portion of a larger study aimed at investigating the effect of a fiber-rich diet containing oat-hulls and daily allocation of roughage on broiler breeder welfare during the rearing period, measured using behavioral, stress physiological, and clinical welfare indicators. A full description of the animals, management, and housing is found in Wurtz et al. (2025).

In summary, day-old female Ross 308 breeder chicks (*N* = 600) from Aviagen (SweHatch AB, Sweden) were housed at an experimental facility at AU Viborg (Tjele, Denmark). Birds were randomly allocated to 12 floor pens in groups of 50 birds at an initial stocking density of 12.5 birds per m^2^. Each pen was bedded with a breeder bedding mix (wood shavings and peat, Spanwall®, Kalundborg, Denmark) and contained a water line with seven nipples with drip cups (Ziggity Systems Inc., Middlebury, Indiana, USA). The litter in all pens was completely refreshed at 11 and 13 weeks of age as litter had degraded in some pens to a point that warranted intervention. Feed was provided on paper on the floor under the water lines for the first week, and thereafter was scattered on the litter by two drop feeders per pen. At 8 days of age, each pen received a pecking block (Vilofoss®, Fredericia, Denmark). At placement, 23 hours of light was provided which was subsequently reduced by 1 hour each day until a light period of 8 h was reached at day 16. Temperature of the room was 33°C at placement and was gradually reduced until 21°C was reached on day 28. From day 28 onward, the temperature was maintained at 20°C, and relative humidity was controlled at 60 % using a negative-pressure ventilation system (SKOV A/S, Glyngøre, Denmark).

Due to instances of cannibalism, light intensity was lowered to 4 lux at around 5 weeks of age and maintained at this level for the remainder of the study. Additionally, a hartshorn oil solution (Vilofarm®, Horsens, Denmark) was applied to birds as a preventative measure. Minorly injured birds were temporarily placed into sick pens for recovery. Any birds with severe injuries were euthanized.

### Experimental treatments

For the first 21 days, all birds received a pelleted commercial “starter 1″ diet. During the first week, the pellets were broken into pieces of approximately 2 mm in size. From 22 days until 42 days of age, birds were fed a pelleted “starter 2″ diet. For the remainder of the study, the birds received a pelleted grower diet. The “starter 2″ diet and grower diets were formulated according to the dietary treatments. Calculated nutrient composition of each treatment is provided in Table 1. Each pen was assigned to one of two dietary treatments: 1) A wheat-based control (CON) diet matching energy content used in commercial diets or 2) A low energy (EXP) diet in which oat hulls were included. Birds received four, three, and two meals per day during the first two, subsequent three, and final two days within the first week period, respectively. Thereafter birds received one meal per day for the remainder of the study which was scattered on the floor at 9:00 h. Maize silage was manually spread across the floor at 9:30 h in the EXP treatment pens. The volume of silage relative to the volume of pelleted feed was gradually increased from 21 days onward and from 56 days of age kept stable at around 10 %. Birds were fed following the growth curve recommended by Aviagen and modified by DanHatch. This meant that birds in each treatment received approximately the same amount of metabolizable energy, though the birds in the EXP treatment received a greater volume of feed due to the inclusion of the fiber. Calculated nutrient composition of each treatment is shown in Table 1.

**Table 1:** Calculated (g/MJ metabolizable energy) and analyzed (g/kg DM) nutrient composition of the control (CON) and experimental (EXP) diets, and maize silage.

	Starter 2 (Day 22-41)		Grower (Day 42-125)		Maize silage	
					Day 22-66	Day 67-125
	CON	EXP	CON	EXP		
Calculated						
ME, MJ ME/kg	11.2	9.74	11.0	9.00		
Macronutrients, g/MJ ME						
Crude protein	1.56	1.56	1.29	1.33		
Crude fat	4.44	4.46	2.84	3.27		
Crude ash	5.71	6.71	5.87	7.41		
Fiber	0.44	1.02	5.61	14.1		
Sugar	2.87	2.85	2.54	2.13		
Starch	33.7	33.8	39.9	32.3		
Amino acids, g/MJ ME						
SID^1^ Lysine	0.60	0.60	0.51	0.51		
SID Methionine	0.44	0.45	0.31	0.33		
SID Cysteine	0.24	0.24	0.20	0.19		
SID Threonine	0.51	0.51	0.44	0.44		
SID Valine	0.61	0.61	0.51	0.51		
SID Tryptophan	0.16	0.16	0.13	0.13		
SID Leucine	0.93	0.94	0.77	0.77		
SID Isoleucine	0.52	0.51	0.42	0.43		
SID Histidine	0.32	0.32	0.26	0.26		
Minerals g/MJ ME						
Calcium	0.85	0.85	0.90	0.99		
Phosphorus	0.69	0.80	0.68	0.70		
Digestible phosphorus	0.03	0.04	0.03	0.03		
Analyzed, g/kg DM						
Dry matter, g/kg	890	900	892	901	354	347
Crude protein, Nx6.25	193	161	156	139	74	69.3
Starch	416	428	428	318	282	298
Sugar	48.3	35.2	39.3	28.6	<9.9	<9.9
Fat	57.0	52.0	41.0	35.0	30.9^2^	30.9^2^
ME energy, MJ/kg^3^	11.1	10.7	10.2	8.13	2.45	2.47

1 Standardized ileal digestible.

2 Literature average based on: Khan et al. (2015), Feedipedia (2016).

3 Calculated ME Poultry: 0,1551 × crude protein + 0,3431 × crude fat + 0,1669 × starch + 0,1301 × total sugar expressed as g/100 g.

### Data collection

#### Clinical Welfare Indicators

At the end of the study (week 18) a welfare assessment was performed by three experienced scorers (10 birds per pen per scorer) on 30 birds randomly selected from each pen. Indicators examined included plumage damage, wounds/scratches, footpad dermatitis, bumblefoot, hock burns, plumage cleanliness, and body weight. Plumage damage was assessed based on the method described in Bilčík and Keeling (1999) in which the body feathers (head, neck, back, rump, covets, under neck, breast, legs, belly) and wing-primary and tail feathers were scored on a six-point scale ranging from 0 (completely intact feathers) to 5 (completely denuded area or almost all feathers missing). Presence of wounds/scratches on the comb and the body (same regions in which plumage damage was assessed) were assessed on a five-point scale, ranging from 0 (no injuries or scratches) to 4 (a wound > 2 cm in diameter) (Bilčík and Keeling, 1999). Footpad dermatitis was assessed using a three-point scale where 0 = no damage, 1 = mild damage (color changes, superficial damage, or dark papillae), 2 = severe damage (wounds or scabs, signs of bleeding or swelling) (Ekstrand et al., 1998). The presence of bumblefoot or hyperkeratosis was also noted on a dichotomous scale (present or absent). Hocks were scored on a four-point scale where a score of 0 = healthy skin with no marks, 1 = redness, 2 = mild scabbing (<10 % of the hock), and 3 = severe scabbing (>10 % of the hock) (Sherlock, et al., 2010). Plumage cleanliness was scored by examining the underside of the bird (i.e., the breast, wings, and thighs). A score of 0 = clean and dry plumage, 1 = slightly dirty or wet and/or discolored plumage (<33 % of the area), 2 = dirty/wet and/or discolored plumage (33-67 % of the area), and 3 = very dirty/wet and/or discolored plumage (>67 % of the area) (Welfare Quality®, 2009). The body weight of each individual was collected prior to euthanasia. Following the assessment, birds were humanely euthanized using CO_2_ gas.

#### Body Weight Uniformity

Birds were weighed on a pen basis upon arrival and weekly throughout the experiment. At four times throughout the experimental period (1, 6, 12, and 18 weeks of age) individual weights were obtained. As weighing was conducted within one to two hours following feeding, the daily feed allowance was subtracted from the pen live weight to account for differences in gut fill between the two treatments (Nielsen et al., 2011; Riber et al., 2021).

#### Fault Bars in Feathers

Following euthanasia, a total of three feathers were collected from each bird for later examination for fault bars. This included one primary wing feather (the left primary 8, i.e., the third outermost wing feather), one primary tail feather (the left rectrix 1, i.e., middle of the tail feathers), and one scapular feather (the left scapular 3, i.e., middle of the scapular feathers). If the required feather was severely broken or dirty, the equivalent feather from the right side was collected instead. Feathers were carefully plucked to maintain completeness of the feather. Feathers were placed in labelled plastic bags and stored in the freezer until fault bars could be analyzed.

After thawing, each feather was macroscopically examined by a single observer for the total number of fault bars, size and location of each fault bar, feather length (mm) and feather weight (mg). For analyses, fault bars were categorized according to their length and severity as follows: 1) minor (< 5 mm), 2) moderate (≥ 5 mm), and 3) severe (≥ 5 mm and broken barbules on the fault bar) (Arrazola and Torrey, 2019).

#### Mortality

Mortality and culls were documented with reasons when known.

#### Litter quality and dry matter content

Every second week (except week 6), beginning from week 2, the quality of the bedding was assessed visually in two locations per pen using the Welfare Quality® scoring system on a scale from 0 to 4, where a score of 0 = completely dry and flaky and a score of 4 = sticks to boots once the compacted crust is broken (Welfare Quality®, 2009). Samples of the bedding were collected from a single location to be analyzed for dry matter content at weeks 6, 12, and 18.

### Statistical analyses

Statistical analysis of clinical welfare indicators, mortality, and litter quality were conducted using SAS version 9.4 (SAS Institute Inc). Analysis of body weight uniformity and fault bar measures were conducted using R Statistical Software v4.3.3 (R Core Team, 2024).

For the analysis of plumage damage, scores for each body location were summed per bird, creating a total body score which could range from 0 (no plumage damage anywhere on the body) to 55 (completely denuded). A mixed effects model was fit with the total body plumage score as the response variable, a fixed effect of dietary treatment, and random effects of observer and pen. Due to too few occurrences, wounds/scratches, footpad dermatitis, and bumblefoot were not formally assessed. The presence of hyperkeratosis was assessed using a binary GLIMMIX procedure with a fixed effect of dietary treatment and random effects of pen and observer. Hock burn score was analyzed using a multinomial generalized mixed model with a fixed effect of dietary treatment, and random effects of pen and observer. A multinomial generalized mixed model was used to assess plumage cleanliness, with a fixed effect of dietary treatment, and random effects of pen and observer.

Body weight was analyzed by fitting a mixed model with response variable of weekly body weight per individual and fixed effects of dietary treatment, age in weeks, and their interaction, and a random effect of pen. Uniformity of body weight was expressed as the percentage of birds within ± 10 % of the mean body weight. The coefficient of variation (CV) was also calculated by dividing the standard deviation by the mean body weight of each pen. Body weight uniformity was analyzed by fitting mixed models with response variables of either body weight uniformity or the CV and fixed effects of dietary treatment and week and a random effect of pen. The effect of week was assessed using a post hoc test with the Kenward-Roger method for calculating degrees of freedom.

For analysis of fault bars, total number of fault bars per feather, total number of severe fault bars per feather, average bar position relative to the base of the feather, and the growth rate (weight and length per week) were analyzed. The growth rate was estimated with the assumption that molting had finished at 12 weeks of age, thus the weight and length of the feathers were divided by six (age when feathers were collected (18 weeks) minus estimated age of molt (12 weeks)). Some of the feathers differed in developmental stage, likely due to being lost and regrown following the molt. These newer feathers were excluded from analyses, apart from the analysis to total fault bars, total severe fault bars, and average bar position for the tail feathers. Newer growth feathers made up roughly half of the collected tail feathers and were statistically similar for number and severity of fault bars, average position of fault bars, and growth rate compared to the older feathers.

The total number of fault bars and the total number of severe fault bars per feather were analyzed using negative binomial mixed-effects models with fixed effects of dietary treatment, feather type (scapula, tail, or wing), and their interaction, and random effects of bird nested within pen. Feather growth, both in terms of length and mass, were analyzed separately for each feather type, as feathers likely molt at different times depending on their body location. Models for growth (length (mm) and mass (mg)) were linear mixed-effects models with fixed effect of dietary treatment and random effect of pen. *P*-values for any post-hoc pairwise comparisons were adjusted using the Tukey-Kramer method. Average fault bar position was analyzed using a linear mixed-effect model with fixed effects of dietary treatment, feather type, and their interaction, and random effect of pen.

Mortality (percentage of each pen) was analyzed non-parametrically using a Wilcoxon test for independent samples with fixed effect of dietary treatment and random effect of pen.

Litter quality was analyzed using Friedmann chi squared tests. Litter dry matter content was analyzed using a mixed model with dietary treatment and age in weeks as fixed effects and pen as a random effect. The significance of fixed effects was assessed using a post hoc Tukey’s test.

## Results

### Clinical welfare indicators

The plumage damage scores for each location for each treatment are presented in Table S1. In both treatments, plumage condition was the poorest on the tail, breast, and belly regions. There was a significant effect of treatment on the total plumage damage score, with birds from the CON treatment having significantly higher scores (i.e., poorer plumage condition) than the EXP treatment (mean ± SE: CON = 15.5 ± 1.70; EXP = 5.8 ± 1.70; *F*_(1,346)_ = 24.25, *P* < 0.0001). Scores for wounds/scratches are presented in Table S2. Occurrences were rare in both treatments and thus no formal analysis was conducted.

**Fig. 1:**
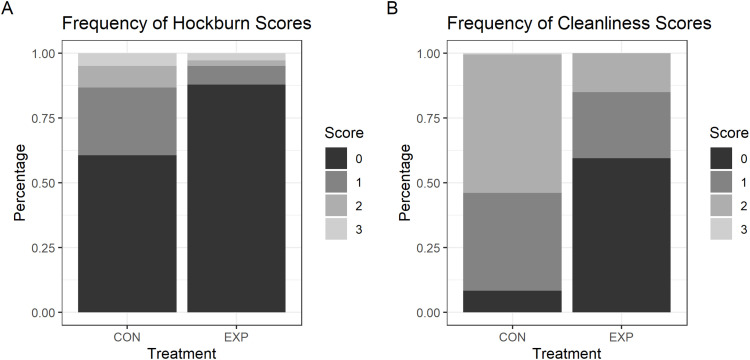
Distribution of hock burn scores (A) and plumage cleanliness scores (B) in the control (CON) and experimental (EXP) treatment groups.

Very few birds (2 %; CON: *n* = 5, EXP: *n* = 2) were observed with footpad dermatitis (Table S3) and thus no further analyses were conducted. Bumblefoot was only observed in one bird in the EXP treatment. A greater number of birds were observed with hyperkeratosis in the CON than the EXP dietary treatment (OR = 8.6, CL = 3.62 - 20.65, *F*_(1,346)_ = 23.75, *P* < 0.0001; Table S4). Birds with hock burns were observed in both treatment groups (Fig. 1A), and the distribution of scores differed between treatments (χ^2^ = 6.56, *P* = 0.0104). Birds in the EXP treatment had higher odds of getting lower (i.e., better) scores (OR = 8.3, CL: 1.64 - 41.68).

Plumage cleanliness scores are shown for both treatments in Figure 1B. The distribution of scores differed between treatments, and birds from the experimental treatment were more likely to have lower scores (i.e., cleaner plumage) (χ^2^ = 7.16, *P* = 0.0075).

### Body weight uniformity

Body weight uniformity within each pen did not differ between the two dietary treatments (*F*_(1,10)_ = 0.15, *P* = 0.7095; Table 2). Week impacted body weight uniformity (*F*_(2,22)_ = 16.83, *P* < 0.0001), with poorer body weight uniformity at week 6 than at weeks 12 and 18 (*P* < 0.0001). CV also did not differ between the two dietary treatments (*F*_(1,10)_ = 0.83, *P* = 0.3827). Week impacted CV of body weight (*F*_(2,22)_ = 6.69, *P* < 0.0054), with week 6 having a higher CV (wider variation) than weeks 12 and 18 (*P* = 0.0017 and 0.0219, respectively).

**Table 2:** Body weight uniformity and the coefficient of variation of body weight during weeks 6, 12, and 18 for the experimental (EXP) and control (CON) dietary treatments. A lower CV means body weights are more similar to the average (i.e., more uniform).

Week	Treatment	Average body weight (g)	Uniformity (%)	CV (%)
6	EXP	736.47	54.08	12.57
6	CON	789.25	53.23	12.68
12	EXP	1687.35	67.88	10.35
12	CON	1606.17	66.65	10.53
18	EXP	2376.03	67.96	11.02
18	CON	2370.62	66.01	11.32

### Fault bars in feathers

The total number of fault bars per feather type is presented for both treatments in Table S5.

A significant interaction between dietary treatment and feather type ($\chi^{2}$ *=* 55.96, *P* < 0.0001) was found for total number of fault bars. Post-hoc pairwise comparisons are presented in Table 3. Birds in both dietary treatments had greater numbers of fault bars on tail feathers compared to both scapula and wing feathers (*P*_adj_ < 0.0001). Birds in the EXP treatment had greater numbers of fault bars on wing feathers than scapula feathers (*P*_adj_ < 0.0001). CON birds had greater numbers of fault bars on scapula feathers compared to EXP birds (*P*_adj_ < 0.0001) but tended to have fewer fault bars on tail feathers compared to EXP birds (*P*_adj_ = 0.068). The number of fault bars on wing feathers did not differ between treatment groups. Table 4

**Table 3:** Post-hoc pairwise comparisons for total number of fault bars by feather type (wing, scapula, and tail) and each dietary treatment (experimental (EXP) and control (CON)). Tests were performed on the log scale and results have been back-transformed. P-values are Tukey-adjusted.

Variable	Comparison	Pairwise ratio	SE	z ratio	P_adj_
Wing	CON vs EXP	1.144	0.091	1.697	0.534
Scapula	CON vs EXP	1.791	0.141	7.389	<0.0001
Tail	CON vs EXP	0.774	0.072	−2.736	0.068
CON	Scapula vs Tail	0.552	0.045	−7.24	<0.0001
	Scapula vs Wing	0.922	0.065	−1.159	0.856
	Tail vs Wing	1.671	0.139	6.179	<0.0001
EXP	Scapula vs Tail	0.239	0.019	−18.273	<0.0001
	Scapula vs Wing	0.589	0.044	−7.099	<0.0001
	Tail vs Wing	2.469	0.193	11.586	<0.0001

**Table 4:** Post-hoc pairwise comparisons for presence of severe fault bars by feather type (wing, scapula, and tail) and each dietary treatment (experimental (EXP) and control (CON)). Tests were performed on the log scale and results have been back-transformed. P-values are Tukey-adjusted.

Variable	Comparison	Pairwise ratio	SE	z ratio	P_adj_
Wing	CON vs EXP	0.994	0.201	−0.029	1.000
Scapula	CON vs EXP	9.329	7.099	2.935	0.039
Tail	CON vs EXP	0.640	0.182	−1.571	0.618
CON	Scapula vs Tail	0.195	0.067	−4.767	<0.0001
	Scapula vs Wing	0.113	0.033	−7.441	<0.0001
	Tail vs Wing	0.582	0.155	−2.032	0.324
EXP	Scapula vs Tail	0.013	0.010	−5.828	<0.0001
	Scapula vs Wing	0.012	0.009	−6.065	<0.0001
	Tail vs Wing	0.903	0.215	−0.429	0.998

An interaction between dietary treatment and feather type was also found for the occurrence of severe fault bars (present/not present) ($\chi^{2}$ *=* 11.055, *P* < 0.004). Birds in both treatments had greater numbers of severe fault bars in their wing and tail feathers compared to their scapula feathers (*P*_adj_ < 0.0001). CON birds had greater counts of severe fault bars in their scapula feathers compared to EXP birds (*P*_adj_ = 0.039).

Tail and scapula feathers grew slower in terms of length in the CON treatment compared to the EXP treatment (tail: *F*_(1,10)_ = 3.679, *P* = 0.0846; scapula: *F*_(1,10)_ = 6.34, *P* = 0.0305). Wing feathers tended to grow slower (length) in the CON treatment compared to the EXP treatment (*F*_(1,10)_ = 4.65, *P* = 0.05731). Growth in terms of mass of the scapula feathers was impacted by dietary treatment, with CON birds tending to have slower growth (*F*_(1,10)_ = 4.192, *P* = 0.0681). Dietary treatment affected average fault bar position relative to the base of the feather (*F*_(1,11)_ = 23.945, *P* = 0.0005), with EXP birds having greater distances.

### Mortality

A total of 28 birds were culled or found dead, and of these 11 were from the EXP treatment and 17 from the CON treatment. See further details in Table S7. No significant difference in mortality between treatments was found. In total, five birds were culled from EXP pens (*n* = 3), whereas 11 were culled from CON pens (*n* = 5) over the course of the study. Of these, one bird from the EXP treatment and five birds from CON treatment pens (*n* = 2) were culled due to injuries resulting from cannibalism. Over the course of the cannibalism outbreak, 38 birds were placed into sick pens temporarily to allow for recovery. Of these birds, all came from pens (*n* = 4) receiving the commercial dietary treatment (CON).

### Litter quality and dry matter content

The visually assessed litter quality in the pens differed significantly between weeks for both treatments in each location (Location 1: CON, Friedmann chi-square= 36.1, DF=7, *P* < 0.0001; EXP: Friedmann chi-square= 35.5, DF=7, *P* < 0.0001 and Location 2: CON, Friedmann chi-square= 38.5, DF=7, *P* < 0.0001; EXP: Friedmann chi-square= 33.1, DF=7, *P* < 0.0001). The litter quality was compared between the two treatments in weeks 8, 10, 12, 14, 16 and 18, and did not differ for either location.

Dry matter content of the litter (Table S8) was affected by a main effect of age in weeks (F_(1,10)_ = 56.36, *P* < 0.0001), with the dry matter content being significantly lower in week 18 compared to both week 6 and 12 (week 6: 94.3 ± 1.20, week 12: 94.6 ± 1.20, week 18: 79.7 ± 1.20; week 18 vs. 6: *P*_adj_ < 0.0001; week 18 vs.12: *P*_adj_ < 0.0001). Treatment tended to affect dry matter content in the litter, being drier in the EXP treatment than in the CON treatment (EXP, 91.1 ± 1.12; CON, 88.0 ± 1.12; *P* = 0.0728).

## Discussion

In a previous study of fast-growing broiler breeders (Tahamtani et al., 2020), it was shown that inclusion of roughage in the diet led to improved plumage condition, while no differences were observed between birds fed a commercial diet or one diluted with oat hulls. Another study found that plumage condition of broiler breeders was better with diets that included soybean hulls and calcium propionate (an appetite suppressant) (Arrazola, et al., 2019), likely indicating a reduction in stereotyped feather pecking. We similarly found improved plumage condition in birds fed a diet high in insoluble fiber and supplemented with roughage. Feather pecking is a complex behavior with both internal motivating factors and external eliciting factors. While little research has been conducted on feather pecking in broiler breeders (De Jong and Guémené, 2011), it is thought that this behavior likely falls under the category of oral stereotypic behavior indicative of chronic hunger and frustrated feeding motivation (Morrissey et al., 2014). Though not formally quantified in our study, feather sucking/licking, a behavior thought to be associated with boredom, nutritional deficiencies, and feed restriction (Taylor, et al., 2024b), was observed in our study and could have further contributed to feather damage. In our study, the increased bulk of the EXP diet combined with the increased foraging opportunities of the scattered roughage may have contributed to a reduction in feather pecking behavior, and thus improved plumage condition. The EXP grower diet did, however, contain a higher concentration of N than anticipated and consequently also a higher concentration per unit of energy. It cannot be excluded that this also led to a higher supplementation of sulfuric amino acids, which is essential for feather development.

While visual litter assessments did not detect differences in litter quality between the two treatments, the analysis of dry matter content did, with pens with birds fed the EXP diet tending to have drier litter. This suggests that the inclusion of fiber in the diet did not increase water in the excreta to an extent to negatively impact litter quality. Further, as the EXP diet contained more bulk, the birds may have spent more time feeding and thus contributing to increased litter turnover which could further explain the drier litter conditions (Ekstrand et al., 1997). Wet litter has consistently been associated with increased prevalence of footpad dermatitis and hock burns (e.g., (Dawkins, et al., 2004; Haslam, et al., 2007; Kaukonen, et al., 2016) and dirty plumage conditions (De Jong, et al., 2014), and likely contributed to the poorer hock condition and plumage dirtiness observed in birds fed the CON dietary treatment. Differences in behavior may have also had an impact, as increased sitting has been shown to contribute to contact dermatitis on the hocks and soiling of the plumage (Göransson, et al., 2020).

Past research has suggested that a greater presence of fault bars and reduced feather growth could be indicative of acute, unpredictable stress such as handling or inconsistent feeding schedules in broiler breeder pullets (Arrazola and Torrey, 2019). In our experiment, CON birds had both a greater number of total fault bars and greater occurrence of severe fault bars on their scapula feathers than EXP birds, possibly suggesting that the birds in the CON treatment experienced greater stress. Arrazola et al. (2019) similarly found a greater number of fault bars in feathers from broiler breeders fed a standard commercial diet compared to those fed an alternative diet diluted with 40 % soybean hulls and the inclusion of calcium propionate as an appetite suppressant. Tahamtani et al. (2020) also observed more fault bars in the scapula feathers in birds fed a standard commercial diet than birds supplemented with roughage, however there were no significant differences between commercial diet birds and those with oat hulls included in the diet. Contrary to results from Tahamtani et al. (2020), we found that EXP birds tended to have more fault bars present on their tail feathers than the CON birds.

The greater number of fault bars present in the tail feathers of EXP birds could be explained by differences in the development of the tail feathers between the two treatments, where more CON birds had smaller, underdeveloped tail feathers (avg. length 79.4 mm) compared to EXP birds (avg. length 92.0 mm). The smaller, underdeveloped tail feathers in the CON birds could possibly have been a result of increased feather pecking resulting in the removal, and subsequent regrowth of the feathers. Additionally, the presence of severe fault bars could have made the feathers more susceptible to breakage, resulting in the observed smaller tail feathers. As severely broken feathers were not collected for the analysis of fault bars, this could have potentially biased the results towards feathers with less severe damage.

Our results indicate that birds fed the CON diet had reduced feather growth compared to those fed the EXP diet. The results in our study were more pronounced than those found by Tahamtani et al. (2020), where they only found a tendency for lower growth rate of the tail feather in terms of length in birds fed a diet diluted with oat hulls compared to a standard commercial diet, and those found by Arrazola et al. (2019) where they found no differences in feather growth between an alternative diet diluted with soybean hulls compared to a standard commercial diet. This could be a result of increased statistical power in our study, or may be due to a greater treatment effect when oat hulls and roughage are provided concurrently.

Body weight uniformity at the end of the study was within an acceptable range (between 10 and 12 %) for both dietary treatments, suggesting that management on farm was appropriate and that uniform flocks may be achieved on the alternative fiber rich diet during the rearing period. Sandilands et al. (2005) similarly found comparable weight uniformity between birds fed an alternative diet diluted with oat hulls and the inclusion of calcium propionate compared to standard basal diets.

Despite the higher incidences of cannibalism in CON pens, total mortality did not differ between the treatments. This is likely due to the quick treatment and preventative measures implemented during the study.

## Conclusion

Results from our study show that feeding a diet high in insoluble fiber and supplemented with roughage appears to provide some improvements to broiler breeder welfare during rearing compared to feeding a standard commercial diet. Birds fed the EXP diet showed improved indicators of welfare such as improved plumage condition, lower incidence and severity of hock burns, fewer fault bars in their scapular feathers, and fewer injuries and culls resulting from cannibalism.

## CRediT authorship contribution statement

**Kaitlin E. Wurtz:** Data curation, Formal analysis, Investigation, Supervision, Writing – original draft, Writing – review & editing. **Karen Thodberg:** Formal analysis, Writing – review & editing. **Anja B. Riber:** Conceptualization, Funding acquisition, Investigation, Methodology, Project administration, Resources, Supervision, Writing – review & editing.

## Disclosures

The authors declare that they have no known competing financial interests or personal relationships that could have appeared to influence the work reported in this paper.

## Appendix Supplementary materials

Image, application 1
