# Supplementary material for: Impact of feeding a high fiber diet and roughage on stress and clinical welfare indicators in fast-growing broiler breeder pullets
Source: Poult Sci. 2025 Oct 8;104(12):105960. doi: 10.1016/j.psj.2025.105960 (PMC12547728; doi:10.1016/j.psj.2025.105960)
Supplement: Supplementary file 1 [file mmc1.docx]

**Impact of feeding a high fiber diet and roughage on stress and clinical welfare indicators in fast-growing broiler breeder pullets**

Kaitlin E. Wurtz^1,2,*^, Karen Thodberg^1^, Anja B. Riber^1^

*^1^Department of Animal and Veterinary Sciences, Aarhus University, DK-8830 Tjele, Denmark*

*^2^Department of Animal Sciences, Purdue University, 270 S. Russell St. West Lafayette, Indiana, USA 47907 (Present address)*

**SUPPLEMENTARY MATERAILS AND METHODS**

**Table S1.** Plumage damage scores by location for the control (CON) and experimental (EXP) treatments.

| **Treatment** | **Variable** | **N** | **Mean** | **Std Dev** | **Min** | **Max** |
| --- | --- | --- | --- | --- | --- | --- |
| **CON** | Head | 180 | 0.2 | 0.44 | 0 | 2 |
|  | Neck | 180 | 0.3 | 0.46 | 0 | 2 |
|  | Back | 180 | 0.5 | 0.72 | 0 | 3 |
|  | Rump | 180 | 1.6 | 1.07 | 0 | 4 |
|  | Covets | 180 | 0.6 | 0.61 | 0 | 3 |
|  | Wings | 180 | 1.5 | 0.65 | 0 | 4 |
|  | Tail | 180 | 3.0 | 0.93 | 0 | 4 |
|  | Underneck | 180 | 0.3 | 0.48 | 0 | 3 |
|  | Breast | 180 | 3.0 | 1.34 | 0 | 4 |
|  | Legs | 180 | 1.5 | 1.10 | 0 | 4 |
|  | Belly | 180 | 3.2 | 1.23 | 0 | 4 |
|  | **Total** | **180** | **15.5** | **4.73** | **5** | **26** |
| **EXP** | Head | 180 | 0.1 | 0.37 | 0 | 3 |
|  | Neck | 180 | 0.1 | 0.29 | 0 | 1 |
|  | Back | 180 | 0.1 | 0.51 | 0 | 3 |
|  | Rump | 180 | 0.3 | 0.56 | 0 | 3 |
|  | Covets | 180 | 0.1 | 0.38 | 0 | 2 |
|  | Wings | 180 | 0.7 | 0.60 | 0 | 3 |
|  | Tail | 180 | 1.2 | 1.06 | 0 | 4 |
|  | Underneck | 180 | 0.0 | 0.21 | 0 | 1 |
|  | Breast | 180 | 1.3 | 1.51 | 0 | 4 |
|  | Legs | 180 | 0.4 | 0.67 | 0 | 3 |
|  | Belly | 180 | 1.4 | 1.52 | 0 | 4 |
|  | **Total** | **180** | **5.8** | **4.84** | **0** | **18** |

**Table S2.** Scores for wounds/scratches by location for the control (CON) and experimental (EXP) treatments.

| **Treatment** | **Variable** | **N** | **Mean** | **Std Dev** | **Min** | **Max** |
| --- | --- | --- | --- | --- | --- | --- |
| **CON** | Head | 180 | 0.00 | 0.000 | 0 | 0 |
|  | Neck | 180 | 0.00 | 0.000 | 0 | 0 |
|  | Back | 180 | 0.00 | 0.000 | 0 | 0 |
|  | Rump | 180 | 0.06 | 0.337 | 0 | 3 |
|  | Covets | 180 | 0.00 | 0.000 | 0 | 0 |
|  | Wings | 180 | 0.00 | 0.000 | 0 | 0 |
|  | Tail | 180 | 0.00 | 0.000 | 0 | 0 |
|  | Underneck | 180 | 0.00 | 0.000 | 0 | 0 |
|  | Breast | 180 | 0.02 | 0.166 | 0 | 2 |
|  | Legs | 180 | 0.00 | 0.000 | 0 | 0 |
|  | Belly | 180 | 0.00 | 0.000 | 0 | 0 |
|  | Comb | 180 | 0.11 | 0.308 | 0 | 1 |
|  | **Total** | **180** | **0.18** | **0.490** | **0** | **3** |
| **EXP** | Head | 180 | 0.00 | 0.000 | 0 | 0 |
|  | Neck | 180 | 0.00 | 0.000 | 0 | 0 |
|  | Back | 180 | 0.00 | 0.000 | 0 | 0 |
|  | Rump | 180 | 0.00 | 0.000 | 0 | 0 |
|  | Covets | 180 | 0.00 | 0.000 | 0 | 0 |
|  | Wings | 180 | 0.00 | 0.000 | 0 | 0 |
|  | Tail | 180 | 0.00 | 0.000 | 0 | 0 |
|  | Underneck | 180 | 0.00 | 0.000 | 0 | 0 |
|  | Breast | 180 | 0.00 | 0.000 | 0 | 0 |
|  | Legs | 180 | 0.00 | 0.000 | 0 | 0 |
|  | Belly | 180 | 0.00 | 0.000 | 0 | 0 |
|  | Comb | 180 | 0.04 | 0.207 | 0 | 1 |
|  | **Total** | **180** | **0.04** | **0.207** | **0** | **1** |

**Table S3.** Prevalence of footpad dermatitis scores (0 = no damage, 1 = mild damage, 2 = severe damage) in the control (CON) and experimental (EXP) treatment groups.

| **Treatment** |  | **Scores** | | | **Total** |
| --- | --- | --- | --- | --- | --- |
|  |  | 0 | 1 | 2 |  |
| **CON** | *N* | 175 | 3 | 2 | 180 |
|  | *%* | 48.6 | 0.8 | 0.6 |  |
| **EXP** | *N* | 178 | 2 | 0 | 180 |
|  | *%* | 49.4 | 0.6 | 0.0 |  |
| **Total** | *N* | 353 | 5 | 2 | 360 |
|  | *%* | 98.1 | 1.4 | 0.6 | 100 |

**Table S4.** Presence (1) or absence (0) of hyperkeratosis observed in the control (CON) and experimental (EXP) dietary treatments.

| **Treatment** |  | **Scores** | | **Total** |
| --- | --- | --- | --- | --- |
|  |  | 0 | 1 |  |
| **CON** | *N* | 137 | 43 | 180 |
|  | % | 38.06 | 11.94 |  |
| **EXP** | *N* | 173 | 7 | 180 |
|  | % | 48.06 | 1.94 |  |
| **Total** | N | 310 | 50 | 360 |
| **Percent** | % | 86.11 | 13.89 | 100 |

**Table S5.** Descriptive stats for the total number of fault bars and total number of severe fault present in both treatment groups by feather type.

| **Treatment** |  |  |  |  |  |  |  |
| --- | --- | --- | --- | --- | --- | --- | --- |
| **CON** | **Variable** | **Mean** | **Std Dev** | **Median** | **Minimum** | **Maximum** | **N** |
| **Scapula** | Total bars | 8.1 | 4.82 | 8.0 | 0.0 | 25.0 | 182 |
|  | Total severe | 0.1 | 0.35 | 0.0 | 0.0 | 2.0 | 182 |
|  | Avg. position | 64.3 | 11.07 | 65.1 | 14.3 | 91.7 | 179 |
|  | Growth (mass) | 5.4 | 1.86 | 5.8 | 1.4 | 8.7 | 182 |
|  | Growth (length) | 14.4 | 1.43 | 14.8 | 8.4 | 16.9 | 182 |
| **Tail** | Total bars | 13.2 | 8.96 | 12.0 | 0.0 | 43.0 | 177 |
|  | Total severe | 0.3 | 1.15 | 0.0 | 0.0 | 11.0 | 177 |
|  | Avg. position | 52.9 | 14.82 | 53.9 | 20.0 | 90.4 | 169 |
|  | Growth (mass) | 10.3 | 5.69 | 8.9 | 2.7 | 45.9 | 176 |
|  | Growth (length) | 13.4 | 3.96 | 13.7 | 4.5 | 26.3 | 177 |
| **Wings** | Total bars | 8.5 | 4.82 | 8.0 | 0.0 | 22.0 | 179 |
|  | Total severe | 0.8 | 1.15 | 0.0 | 0.0 | 6.0 | 179 |
|  | Avg. position | 82.4 | 11.96 | 82.2 | 48.5 | 115.2 | 173 |
|  | Growth (mass) | 35.3 | 11.08 | 32.5 | 26.3 | 87.1 | 179 |
|  | Growth (length) | 24.6 | 1.67 | 24.9 | 17.3 | 29.7 | 179 |
| **EXP** | **Variable** | **Mean** | **Std Dev** | **Median** | **Minimum** | **Maximum** | **N** |
| **Scapula** | Total bars | 4.6 | 3.46 | 4.0 | 0.0 | 16.0 | 184 |
|  | Total severe | 0.0 | 0.10 | 0.0 | 0.0 | 1.0 | 184 |
|  | Avg. position | 69.8 | 11.38 | 70.8 | 18.8 | 96.3 | 170 |
|  | Growth (mass) | 6.0 | 1.94 | 6.9 | 1.5 | 9.4 | 184 |
|  | Growth (length) | 15.1 | 1.32 | 15.5 | 8.4 | 17.1 | 184 |
| **Tail** | Total bars | 16.7 | 10.87 | 16.0 | 0.0 | 54.0 | 179 |
|  | Total severe | 0.5 | 1.75 | 0.0 | 0.0 | 13.0 | 179 |
|  | Avg. position | 61.6 | 16.65 | 62.2 | 22.6 | 98.5 | 173 |
|  | Growth (mass) | 12.5 | 6.06 | 11.2 | 2.9 | 34.3 | 179 |
|  | Growth (length) | 15.5 | 4.28 | 15.7 | 5.5 | 24.1 | 179 |
| **Wings** | Total bars | 7.0 | 4.68 | 7.0 | 0.0 | 21.0 | 181 |
|  | Total severe | 0.8 | 1.32 | 0.0 | 0.0 | 8.0 | 181 |
|  | Avg. position | 85.4 | 13.36 | 87.8 | 40.1 | 112.4 | 170 |
|  | Growth (mass) | 36.1 | 13.27 | 32.2 | 22.1 | 99.7 | 181 |
|  | Growth (length) | 24.8 | 2.49 | 25.4 | 13.2 | 29.5 | 181 |

**Table S6.** Distribution of numbers of birds with either severe fault bars present or not present (numbers and percentage) on each feather type within the control (CON) and experimental (EXP) treatments.

|  |  | **CON** | |  | **EXP** | |  |
| --- | --- | --- | --- | --- | --- | --- | --- |
| **Feather** |  | Not present | Present | Total | Not present | Present | Total |
| **Scapula** | N | 167 | 15 | 182 | 182 | 2 | 184 |
|  | % | 91.8 | 8.2 | 100.0 | 98.9 | 1.1 | 100.0 |
| **Tail** | N | 158 | 19 | 177 | 154 | 25 | 179 |
|  | % | 89.3 | 10.7 | 100.0 | 86.0 | 14.0 | 100.0 |
| **Wing** | N | 93 | 86 | 179 | 107 | 74 | 181 |
|  | % | 52.0 | 48.0 | 100.0 | 59.1 | 40.9 | 100.0 |

**Table S7.**  Mortality, expressed as percentage culled or found dead from each pen, for the control (CON) and experimental (EXP) dietary treatments.

| **Treatment** | **N** | **Mean** | **Std Dev** | **Median** | **Min** | **Max** |
| --- | --- | --- | --- | --- | --- | --- |
| **CON** | 6 | 5.7 | 4.08 | 6 | 0 | 10 |
| **EXP** | 6 | 3.7 | 2.94 | 3 | 0 | 8 |

**Table S8.** Descriptive stats for dry matter content for the control (CON) and experimental (EXP) treatments by week of testing.

|  | Week | N | Mean | SD | Median | Q25 | Q75 | Min | Max |
| --- | --- | --- | --- | --- | --- | --- | --- | --- | --- |
| EXP | 6 | 6 | 94.6 | 1.25 | 95 | 94 | 96 | 93 | 96 |
|  | 12 | 6 | 95.3 | 2.21 | 95 | 93 | 98 | 93 | 98 |
|  | 18 | 6 | 83.3 | 6.85 | 82 | 77 | 88 | 77 | 93 |
| CON | 6 | 6 | 94.0 | 3.53 | 95 | 94 | 96 | 87 | 96 |
|  | 12 | 6 | 93.9 | 1.88 | 94 | 93 | 95 | 92 | 97 |
|  | 18 | 6 | 76.2 | 4.90 | 75 | 73 | 80 | 70 | 83 |
